# Standardized evaluation of the extent of resection in glioblastoma with automated early post-operative segmentation

**DOI:** 10.3389/fradi.2024.1357341

**Published:** 2024-05-22

**Authors:** Lidia Luque, Karoline Skogen, Bradley J. MacIntosh, Kyrre E. Emblem, Christopher Larsson, David Bouget, Ragnhild Holden Helland, Ingerid Reinertsen, Ole Solheim, Till Schellhorn, Jonas Vardal, Eduardo E. M. Mireles, Einar O. Vik-Mo, Atle Bjørnerud

**Affiliations:** ^1^ Computational Radiology and Artificial Intelligence (CRAI), Department of Physics and Computational Radiology, Clinic for Radiology and Nuclear Medicine, Oslo University Hospital, Oslo, Norway; ^2^ Department of Physics, University of Oslo, Oslo, Norway; ^3^ Department of Physics and Computational Radiology, Clinic for Radiology and Nuclear Medicine, Oslo University Hospital, Oslo, Norway; ^4^ Department of Radiology, Clinic for Radiology and Nuclear Medicine, Oslo University Hospital, Oslo, Norway; ^5^ Department of Medical Biophysics, University of Toronto, Toronto, ON, Canada; ^6^ Sandra E Black Centre for Brain Resilience and Recovery, Sunnybrook Research Institute, Toronto, ON, Canada; ^7^ Institute of Clinical Medicine, Faculty of Medicine, University of Oslo, Oslo, Norway; ^8^ Department of Neurosurgery, Oslo University Hospital, Oslo, Norway; ^9^ Department of Health Research, SINTEF Digital, Trondheim, Norway; ^10^ Department of Circulation and Medical Imaging, Norwegian University of Science and Technology (NTNU), Trondheim, Norway; ^11^ Department of Neurosurgery, St. Olavs Hospital, Trondheim University Hospital, Trondheim, Norway; ^12^ Department of Neuromedicine and Movement Science, Norwegian University of Science and Technology (NTNU), Trondheim, Norway; ^13^ Center for Lifespan Changes in Brain and Cognition, University of Oslo, Oslo, Norway

**Keywords:** segmentation, glioblastoma, MRI, deep-learning, RANO, extent of resection, early post-operative, overall survival

## Abstract

Standard treatment of patients with glioblastoma includes surgical resection of the tumor. The extent of resection (EOR) achieved during surgery significantly impacts prognosis and is used to stratify patients in clinical trials. In this study, we developed a U-Net-based deep-learning model to segment contrast-enhancing tumor on post-operative MRI exams taken within 72 h of resection surgery and used these segmentations to classify the EOR as either maximal or submaximal. The model was trained on 122 multiparametric MRI scans from our institution and achieved a mean Dice score of 0.52 ± 0.03 on an external dataset (*n* = 248), a performance ­on par with the interrater agreement between expert annotators as reported in literature. We obtained an EOR classification precision/recall of 0.72/0.78 on the internal test dataset (*n* = 462) and 0.90/0.87 on the external dataset. Furthermore, Kaplan-Meier curves were used to compare the overall survival between patients with maximal and submaximal resection in the internal test dataset, as determined by either clinicians or the model. There was no significant difference between the survival predictions using the model's and clinical EOR classification. We find that the proposed segmentation model is capable of reliably classifying the EOR of glioblastoma tumors on early post-operative MRI scans. Moreover, we show that stratification of patients based on the model's predictions offers at least the same prognostic value as when done by clinicians.

## 1 Introduction

Glioblastoma is the most common malignant primary brain tumor in adults (1). It is also the most aggressive brain tumor, with a median overall survival of 14–15 months despite comprehensive treatment including surgical resection and subsequent chemotherapy and radiotherapy (2).

Magnetic resonance imaging (MRI) is the diagnostic tool of choice for diagnosis, surgical planning, and follow-up management. Post-operative imaging plays a key role in evaluating the extent of resection (EOR), which is the extent to which the tumor is removed during surgery. Because higher EOR of the contrast-enhancing tumor (CET) correlates with improved prognosis (3), classifications based on the EOR are frequently used to stratify patients in clinical trials evaluating treatment outcomes and novel therapies for glioblastoma (4–6). The latest response assessment in neuro-oncology (RANO) guidelines, seeking to standardize practices, recommend using a 1 ml cut-off to classify patients into *maximal CET resection* if the volume of the CET remaining after surgery is ≤1 ml, or *submaximal CET resection* if >1 ml remains (7). This classification is found to offer the best prognostic value.

Differentiating between maximal and submaximal CET resection requires volumetric segmentation of the CET on post-operative MRI. The segmentation should be done on early post-operative MRI, preferably within 48–72 h of surgery. Scans acquired at later time points may show late post-operative reactive changes and contrast leakage in the brain parenchyma or disease progression, due to the aggressive nature of the tumor, which could lead to an overestimation of the residual CET volume (8). Segmenting the CET on early post-operative images, unfortunately, is not part of most hospitals’ current clinical practice, as it is time-consuming and adds to the strain of radiology departments. Moreover, segmenting post-operative CET is inherently challenging, showing poor interrater agreement even between experienced radiologists (9).

Automatic segmentation models could provide a reproducible measure of the EOR without imposing additional workload on radiologists. Pre-operative glioblastoma segmentation has recently improved dramatically, in large part due to the success of deep-learning approaches (10–12) and the availability of public data repositories, including the Multimodal Brain Tumor Segmentation (BraTS) dataset (13–15). These factors have also led to advances in post-operative segmentation at follow-up (weeks to months after surgery) (16, 17). For early post-operative segmentation, previous research has predominantly focused on semi-automatic methods that require user input, and thus fail to scale to large datasets (18–20). There has, however, been a shift towards fully automated methods, starting with a study by Meier et al. that developed a segmentation method using random forest classification of features extracted from 19 patients (21). More recently, Bianconi et al. trained a deep-learning segmentation model on a dataset that included 71 early post-operative images (22), while Helland et al. utilized a large dataset of 956 early post-operative images to train separate deep-learning models (23). However, these studies are predominantly technical in nature and fall short of confirming the clinical utility of the models due to the omission of clinical endpoints in their evaluations.

In the current study, we aim to assess whether a nnU-Net-based model is capable of segmenting glioblastoma on early post-operative MRI in a clinically meaningful way. Following the training of the model with a semi-supervised technique, we evaluate it by involving clinicians in rating the clinical utility of the resulting segmentations, as well as using standard segmentation metrics. Moreover, we obtain an objective and clinically relevant evaluation by comparing the median overall survival in patient groups stratified by EOR using either the model or clinical assessment. To the best of our knowledge, we are the first to include clinician input and use survival data to evaluate a segmentation model in this medical context. The model, along with the corresponding inference code and a pipeline for streamlined inference, is openly available.^1^

## 2 Materials and methods

### 2.1 Description of the datasets

Two datasets are used in the current study: (1) an internal dataset used for model training and internal testing, and (2) an independent dataset from a collaborating, national institution, which we denote as the external dataset.

Data collection for the internal dataset was based on a retrospective cohort of glioblastoma patients at our institution. All patients undergoing first-time surgery were prospectively registered since 2003 at our institutional quality-control database. In this study we have included all patients who (1) were diagnosed with a histopathologically verified supratentorial GBM (2003–2016), GBM WHO grade IV (2016–2019), or tumors classified as gliosarcoma, giant cell GBM, or epithelioid GBM, according to the relevant WHO classification of tumors of the central nervous system at the time (24); (2) had undergone surgical resection of the tumor between 2003 and 2020 and (3) had a postoperative MRI scan taken within 72 h following surgery that included T1-weighted scans taken before (T1w) and after intravenous injection of a Gadolinium-based contrast (T1wc), T2-weighted (T2w), and T2-weighted fluid-attenuated inversion recovery (T2-FLAIR) scans. A total of 616 patients were included in this study. The cohort had a mean age at surgery of 59.6 (SD 12.4) years and a male-to-female ratio of 1.34 (56 patients had missing sex information).

The external dataset consisted of early post-operative MRIs of a total of 248 patients with glioblastoma and was a subset of the data reported in a previous study (25). All patients in this dataset underwent first-time resection surgery for histologically verified glioblastoma between 2007 and 2020. In the period from 2007 to 2016 the 2007 WHO classification of central nervous system tumors was used, from 2016 to 2020 the 2016 WHO classification was used. Availability of post-operative imaging was the same as for the internal dataset.

Table 1 shows the distribution of MR images used in the internal and external dataset according to MRI manufacturer, field strength, and type of acquisition. Exams in the external dataset were on average acquired at higher field strengths compared to exams in the internal dataset. The use of volumetric (3D) acquisition also differed between datasets.

**Table 1:** Description of the internal and external datasets in terms of scanner manufacturer and field strength, as well as the number of exams per sequence where volumetric (3D) acquisitions (vs. multi-slice 2D) were used.

Dataset	Internal (616 exams)	External (248 exams)
Manufacturer		
Siemens	556 (90%)	215 (87%)
Philips	45 (7%)	33 (13%)
GE	15 (2%)	0
Field strength		
0.95T	23 (4%)	0
1.5T	509 (82%)	140 (56%)
3T	84 (14%)	108 (44%)
3D Acquisition		
T1w	302 (49%)	38 (15%)
T1wc	354 (57%)	235 (95%)
T2-FLAIR	220 (36%)	37 (15%)
T2	0	0

### 2.2 Data subsets

**Figure 1:**
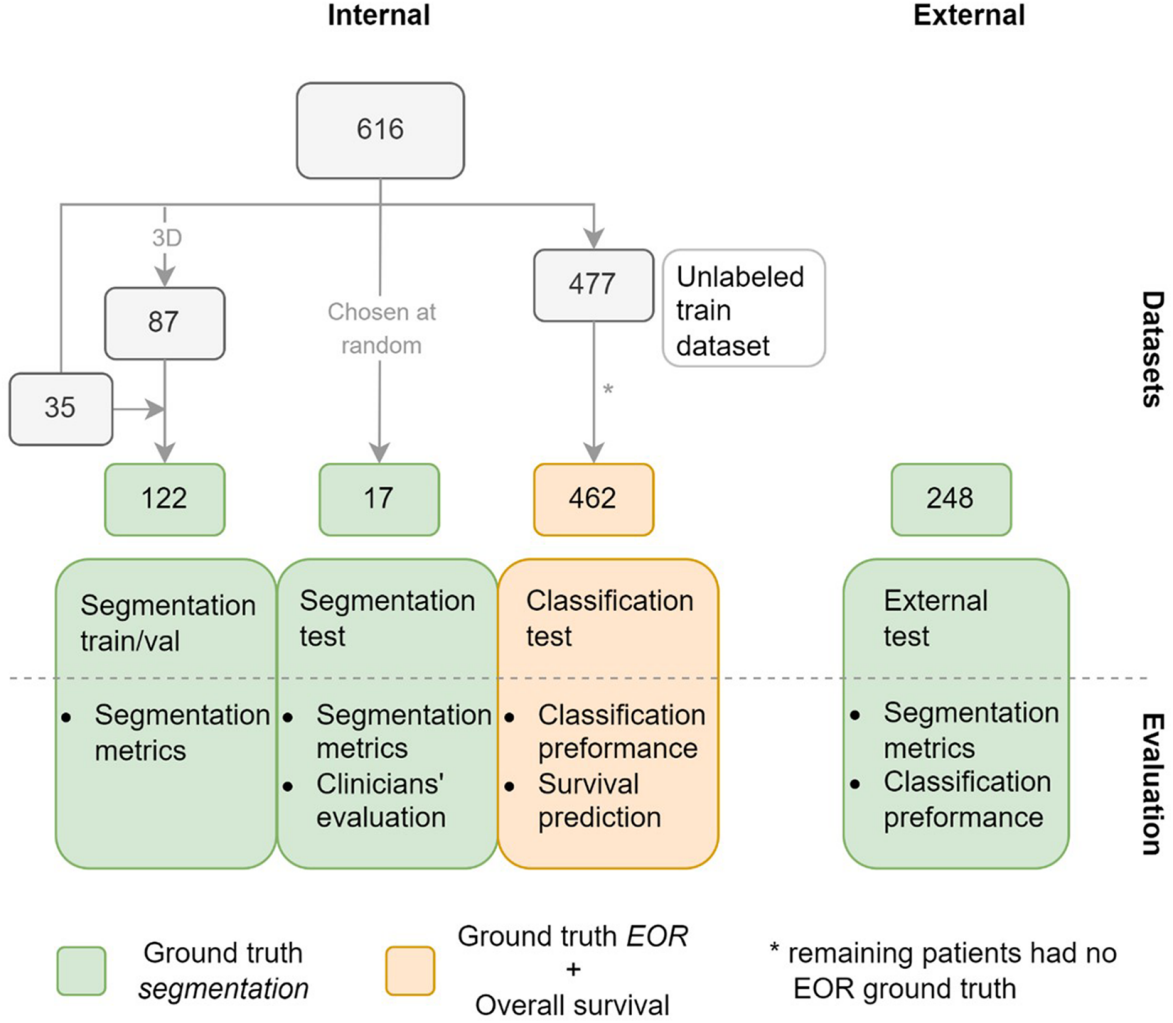
Datasets used in the study with the number of patients in each subset. Green indicates that ground truth (GT) segmentations are available for the subsets, orange that GT extent of resection (EOR) classifications and overall survival time are available for the subset. The figure also shows how the datasets are used to evaluate the model.

The labeled train/validation subset consisted of 122 exams sampled from the internal dataset, as shown in Figure 1. Most of these, 87 exams, were selected based on two criteria: data acquisition between 2016 and 2020; and T1w, T1wc, and T2-FLAIR being 3D acquisitions. As a result, the train/validation subset was skewed towards higher-resolution MRIs compared to the complete dataset. This was done by design to maximize the quality of the training data. The remaining 35 scans were mostly older 2D acquisitions with existing annotations from a previous study.

Our study used two separate internal test subsets, one to test the accuracy of the model's segmentations (segmentation test subset), and another to test the performance of the EOR classification derived from the model's segmentations (classification test subset). The segmentation test subset consists of 17 exams randomly selected from the internal dataset. Note that this subset differs from the train/validation subset in that it was not chosen to maximize the quality of the data. Of the 477 patients not included in either of the aforementioned subsets, 462 had exams with a ground truth (GT) EOR classification available and formed the classification test subset. The external dataset in its entirety formed the third and final test dataset.

### 2.3 Data pre-processing

Pre-processing of the datasets included bias field correction, resampling to 1mm^3^ isotropic voxel size, and within-subject affine registration to the T1wc scan using the Advanced Normalization Tools (ANTs) software.^2^ Lastly, the brain extraction tool HD-BET (26) was used to remove the skull.

### 2.4 Data annotation

Two forms of labeling were carried out on the internal dataset, as shown in Figure 1: outlining GT segmentations, which was done for the train/validation and the segmentation test subsets, and labeling the exams in the classification test subset as showing either maximal or submaximal CET resection.

In total, 387 GT segmentations were used in this study: 122 for training and validation, 17 for internal testing, and 248 for external testing. The GT segmentations outlined specifically for this study, 87 for the train/validation subset and 17 for the segmentation test subset, were annotated by a neuroradiologist with 9 years of experience. The annotator was tasked with outlining the CET, which was defined as high signal tissue on T1wc exams, while avoiding other post-operative findings such as blood products, pneumocephalus or the resection cavity. Note that all four sequences were available to facilitate the differentiation between CET and other post-operative findings. To produce the GT segmentations, the annotator used ITK-SNAP^3^ to edit the preliminary segmentations provided to facilitate the annotation procedure. At the beginning of the annotation process, the preliminary segmentations were generated using a deep-learning model trained exclusively on the 2019 BraTS dataset (15). Later, as GT segmentations became available, the model was fine-tuned using these GTs and used to generate the preliminary segmentations for the data awaiting annotation. These GT segmentations were outlined on the exams after pre-processing of the dataset. The remaining 35 GT segmentations used in the train/validation subset had been previously outlined by a radiologist with 5 years of experience. This annotator was also tasked with outlining the CET using ITK-SNAP while avoiding post-operative findings, however, no preliminary segmentations were provided. Note also that these GT segmentations were outlined on the exams in the original resolution and were later resampled to 1mm^3^ isotropic voxel size, using nearest neighbor interpolation, to conform to the rest of the dataset. The 248 exams in the external dataset were annotated by trained annotators under the supervision of neuroradiologists and neurosurgeons. These annotations were also done on the exams in the original resolution.

All 462 patients in the classification test subset were classified according to the EOR. For most of the patients, the EOR was extracted from the post-operative MRI radiology report. If no CET was seen in the early post-operative exam, the surgery was classified as maximal CET resection. If residual CET was identified in the exam, the surgery was classified as submaximal CET resection. For the patients without an available radiological report, the classification was done by a neurosurgeon not involved in the surgery, following the same criteria. Note that this classification did not use the 1 ml threshold that the newest guidelines recommend, as manual segmentation was deemed to be unfeasible. Survival data were available for patients in this subset.

### 2.5 Network architecture and training procedure

**Figure 2:**
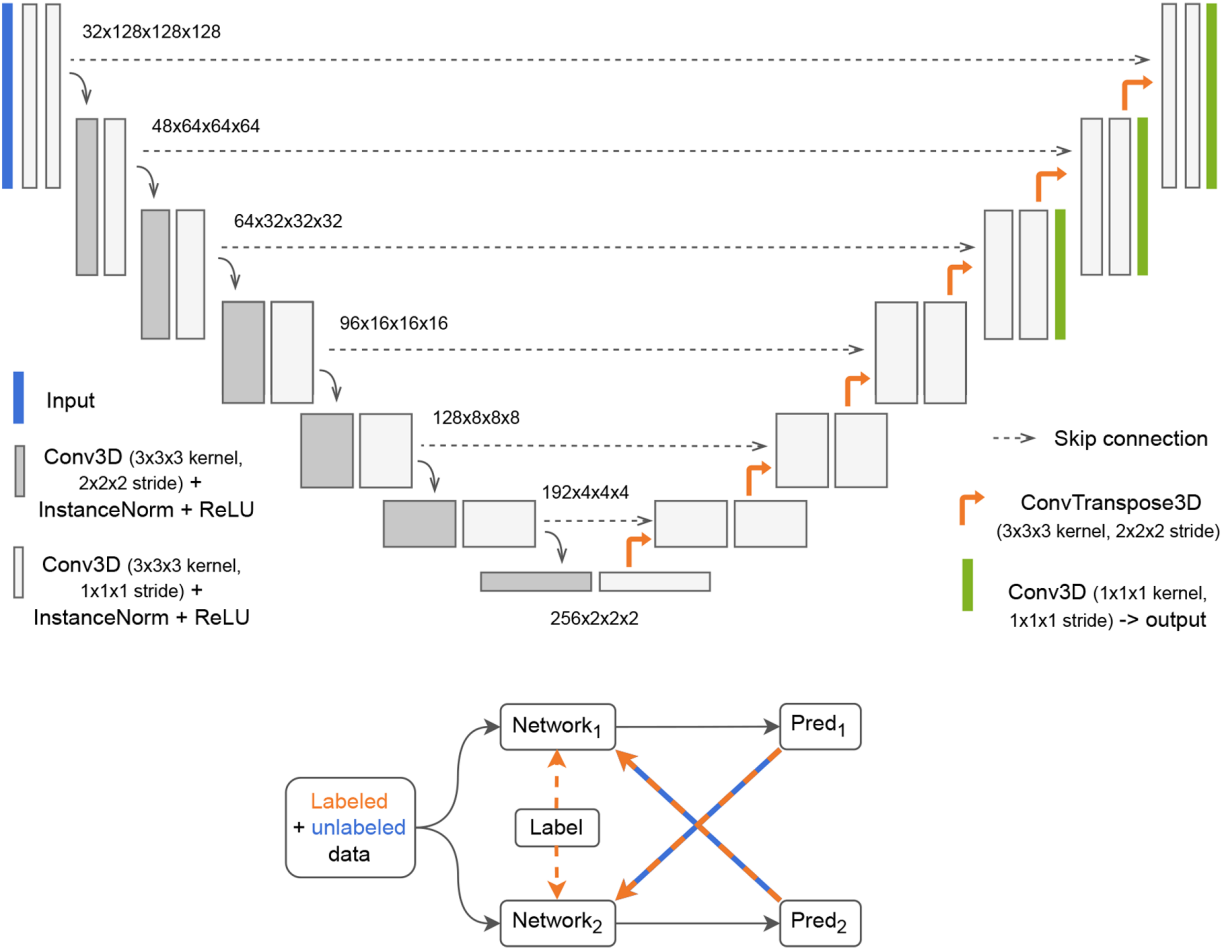
Top: 3D nnU-Net network with deep supervision. The input to the network is a tensor with four channels, one for each scan volume (T1-weighted with and without contrast, T2-weighted and fluid attenuated inversion recovery). The output is a binary mask. Bottom: Cross-Pseudo Supervision (CPS) is a semi-supervised training scheme where labeled and unlabeled data are passed to two networks. For the labeled data, the loss is calculated as a weighted sum of the Dice between each network's prediction and the label (supervised loss) and the Dice between each network's prediction and the other network's prediction (unsupervised loss). For the unlabeled samples, only the unsupervised loss is used.

A U-Net-based (27) deep learning architecture was used in this study. Variants of the U-Net architecture have consistently shown strong performance in medical imaging tasks (12, 28). One such variant, denoted nnU-Net, achieved state-of-the-art performance in the BraTS challenge (12) which involved pre-operative MRI segmentation of glioblastoma and low-grade gliomas. We used the Medical Open Network for Artificial Intelligence (MONAI)^4^ framework to implement a close approximation of the nnU-Net network, configured as illustrated in Figure 2. The Dice score was calculated for the whole volume and defined as the training loss function. The last three layers of the network were used to calculate a deep supervised loss function. However, such a network trained in a standard fully-supervised manner cannot learn from unlabeled data. To use the entirety of our data, which contains over 80% unlabeled data (without GT segmentations), we implemented a semi-supervised learning technique called cross-pseudo-supervision (CPS) (29) using the nnU-Net-architecture as the backbone. The method consists of two nnU-Nets with different initializations that are trained jointly (see Figure 2). To help convergence, each network was pre-trained separately on the BraTS dataset before being jointly trained with CPS. During the CPS training stage, each network was trained in a supervised manner by relying on the predicted pseudo-label from the other network. For the labeled samples, the GT labels were also included in the calculation of the loss function. CPS has been shown to improve performance by 3%–4% from the supervised baseline both on non-medical (29) and medical (30) segmentation tasks. The current implementation closely resembles the work in (30).^5^ The predicted segmentations on both the internal and the external test datasets were obtained from an ensemble of the five models trained with five-fold cross-validation using the labeled train/validation subset as well as the unlabeled train subset.

The models were trained for 500 epochs using the Adam optimizer with weight decay set to 2e-5 and a cosine annealing scheduler with linear warmup with a top learning rate of 3e-4. Training each model took approximately 34 h (hardware specification: NVIDIA A100 40GB GPU). A mini-batch size of 4 was used, including 2 unlabeled samples, and each sample was randomly cropped to 128 × 128 × 128 during training. At inference, a sliding window with overlapping patches ensured the entire volume was segmented.

### 2.6 Data augmentation

Data augmentation is critical to ensure that a model is robust to domain shifts between training and test data. While our training subset contained mostly 3D-acquired MRI exams, our test subset was sampled from the entire dataset, with most scans acquired in 2D. To bridge this domain shift and ensure generalization, the 3D-acquired scans were downsampled during training. With a probability of 0.5, a scan acquired in 3D was downsampled to a randomly chosen number of slices between 20 and 60 (approximately 2–6 mm slice thickness) along an orthogonal orientation chosen at random (coronal, sagittal or axial). Additionally, common data augmentation techniques were employed as implemented in MONAI. Spatial transforms included random flipping, rotation, and zooming, while pixel-wise augmentations were used to randomly modify contrast, shift the intensity histogram, and scale the intensity.

### 2.7 Evaluation of the predictions

We evaluated the model through four experiments, as shown in Figure 1: segmentation metrics, clinician's subjective evaluations of the segmentations, classification metrics to assess the model's performance in classifying the EOR, and performance in predicting overall survival.

#### 2.7.1 Segmentation metrics

The segmentation metrics used were the Dice score and the 95th percentile Hausdorff distance (HD95). The Dice score measures the voxel-wise overlap between the predicted and the GT segmentation, with 0 being no overlap (including the case where either the predicted or the GT segmentation is empty) and 1 being complete overlap. Because the HD95 measures a distance between two segmentations, it is undefined when either the predicted or the GT segmentation is empty. We excluded these undefined cases when computing the HD95. The mean Dice and HD95 with 95% confidence intervals were calculated for the train/validation subset (using 5-fold cross-validation), the segmentation test subset, and the external dataset.

#### 2.7.2 Clinician evaluations

To capture subjective preferences, three clinicians independently evaluated the 17 predicted segmentations and the corresponding 17 GT segmentations in the segmentation test subset. The three clinicians were blinded in this evaluation and rated the 34 segmentations in random order. The raters were instructed to use a scale from 1 to 5, where 1 indicated that the segmentation had no clinical value and 5 was a perfect segmentation that did not require manual refinement. A neuroradiologist with 19 years of experience and two neurosurgeons with 19 and 5 years of experience performed the ratings. The Wilcoxon signed-rank test at a significance level of *p* = 0.05 was used to compare the predicted segmentations against the GT segmentations, with the null hypothesis being that the median difference between the two groups was zero. We used the intra-class correlation coefficient (ICC) (31) to quantify inter-rater agreement in the ratings of the segmentations. Specifically, the ICC form ICC(2,1) was used.

#### 2.7.3 EOR classification performance

We measured the model's classification performance by comparing the EOR classification derived from the predicted segmentations with the GT EOR classification. Unlike the classification test subset, the external dataset did not include GT EOR classifications obtained from radiological reports. Instead, the GT segmentation volumes thresholded at 1 ml were used to classify the scans as showing either maximal or submaximal CET resection, establishing the GT EOR classification for the external dataset. The predicted segmentations were binarily classified, using varying thresholds, as either maximal CET resection if the predicted volume was less than the threshold, or submaximal CET resection otherwise. Thus, we could calculate the receiver operating characteristic (ROC) curve, which shows the sensitivity and specificity at different thresholds of the predicted tumor volume. From the ROC curve we obtained the area under the curve (AUC). Additionally, following the latest recommendations (7), we set the threshold of the predicted volume to 1 ml and calculated the confusion matrix for that threshold as well as the precision and recall values. Note that while previous guidelines called for including the relative reduction in tumor volume when classifying the EOR (32), using only the volume of the remaining tumor has been shown to provide the same prognostic value (7).

#### 2.7.4 Survival prediction

Kaplan–Meier curves were used to compare the overall survival between patients with maximal and submaximal CET resection, as determined by clinicians and the model. The log-rank test at a significance level of *p* = 0.05 assessed survival differences between patients with EOR classified by clinicians and the model.

## 3 Results

After comparing the distributions in the GT annotations between the subsets, we evaluated the model using segmentation metrics, clinician's subjective evaluations, the model's EOR classification performance and survival data. Lastly, example cases illustrated the model's strengths and limitations in accurately predicting CET.

### 3.1 Comparison of data subsets

There are differences in the mean GT volumes between the data subsets used in this study. As depicted in Table 2, the mean CET volume of the train/validation subset is considerably lower than that of the segmentation test subset. The external test dataset has the highest mean CET volume, almost 2.5 times that of the train/validation subset. These volume differences reflect on the GT EOR of the subsets (calculated using the 1 ml threshold), with 37% of patients in the train/validation subset classified as showing submaximal resection vs. 76% in the segmentation test subset and 54% in the external dataset. In the classification test subset, where the GT EOR classification was obtained from radiological reports, submaximal resection was seen in 71% of patients.

**Table 2:** Average dice score and 95th percentile hausdorff distance (HD95), as well as average volumes of the predicted and the GT segmentations for all datasets with GT segmentations. Non-empty means that exams with empty GT segmentations were excluded. The 95% confidence intervals are reported.

Dataset	Train/validation (122 exams)	Segmentation test (17 exams)	External test (248 exams)
Metric			
Dice	0.49 ± 0.05	0.64 ± 0.11	0.36 ± 0.04
Dice non-empty	0.51 ± 0.05	0.64 ± 0.11	0.52 ± 0.03
HD95 [mm]	16 ± 3	20 ± 10	16 ± 2
Segmentation volume [ml]			
GT	1.77 ± 0.54	3.10 ± 1.25	4.40 ± 0.97
Prediction	1.79 ± 0.53	2.60 ± 0.99	2.63 ± 0.46
GT, non-empty	1.85 ± 0.55	3.10 ± 1.25	6.38 ± 1.30
Prediction, non-empty	1.87 ± 0.55	2.60 ± 0.99	3.44 ± 0.59

### 3.2 Segmentation metrics

The segmentation metrics are shown in Table 2. The mean Dice was lower for the external than for the internal dataset. However, the external dataset also had the highest proportion of cases where no residual tumor was found by the annotator (empty GT segmentations), 31% vs. 4% in the train/validation and none in the segmentation test subset. The mean Dice that excluded patients with empty GT segmentations, and thus accounted for this discrepancy, was similar for both datasets: 0.51 and 0.52 for the internal train/validation subset and the external dataset respectively. The mean HD95 was 16 mm for both datasets. The highest Dice, but also the poorest HD95, was reported on the segmentation test subset. This was also the smallest dataset, with only 17 samples vs. 122 and 248 for the train/validation subset and the external dataset respectively.

Table 2 also shows the mean volumes of the predicted and GT segmentations. We show the mean volumes of all samples as well as all samples excluding those with empty GT segmentations. The model underestimated the volume in the external dataset (all samples) by 1.76 ± 0.63 ml and underestimated the volume in the segmentation test subset by 0.50 ± 0.46 ml. For the train/validation subset, the mean predicted volume was 0.02 ± 0.12 ml larger than the mean GT volume.

### 3.3 Clinician evaluations

**Figure 3:**
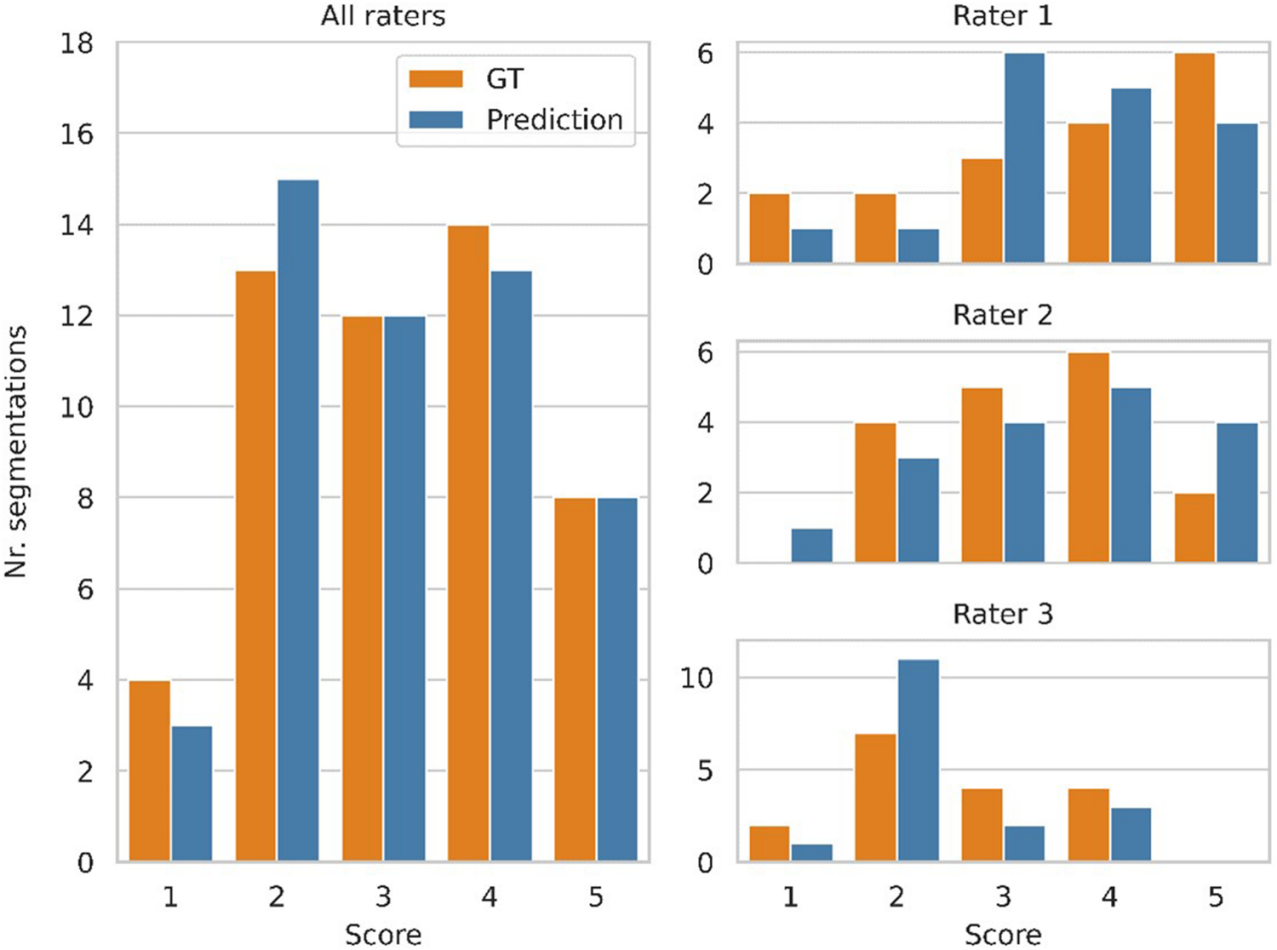
Clinician ratings, with a score of 5 indicating a perfect segmentation that requires no manual refinement, while a score of 1 refers to a segmentation that has no clinical value. Three clinicians scored 17 segmentations produced by the model and the corresponding ground truth segmentations, while being blinded to which group each segmentation belonged to. The plots on the right show the distribution of scores from each clinician. The distribution of all scores (combined from all three clinicians) is shown on the left.

Figure 3 shows a histogram of the clinicians’ subjective evaluations, with each datapoint corresponding to one clinician's score for one segmentation. There was no significant difference (*p* = 0.41) between the clinicians’ ratings for the predicted and GT segmentations, with a mean rating of 3.16 vs. 3.18 respectively. There was however substantial interrater disagreement, with an ICC of 0.21, which is considered poor (33). For about half of the GT segmentations the ratings of two clinicians differed by more than one point, and the same was the case for the predicted segmentations. 13 predicted segmentations and 10 GT segmentations were given a low score (1 or 2) by at least one rater, while 2 predicted segmentations and 3 GT segmentations were given a high score (4 or 5) by all three raters.

### 3.4 EOR classification performance

**Figure 4:**
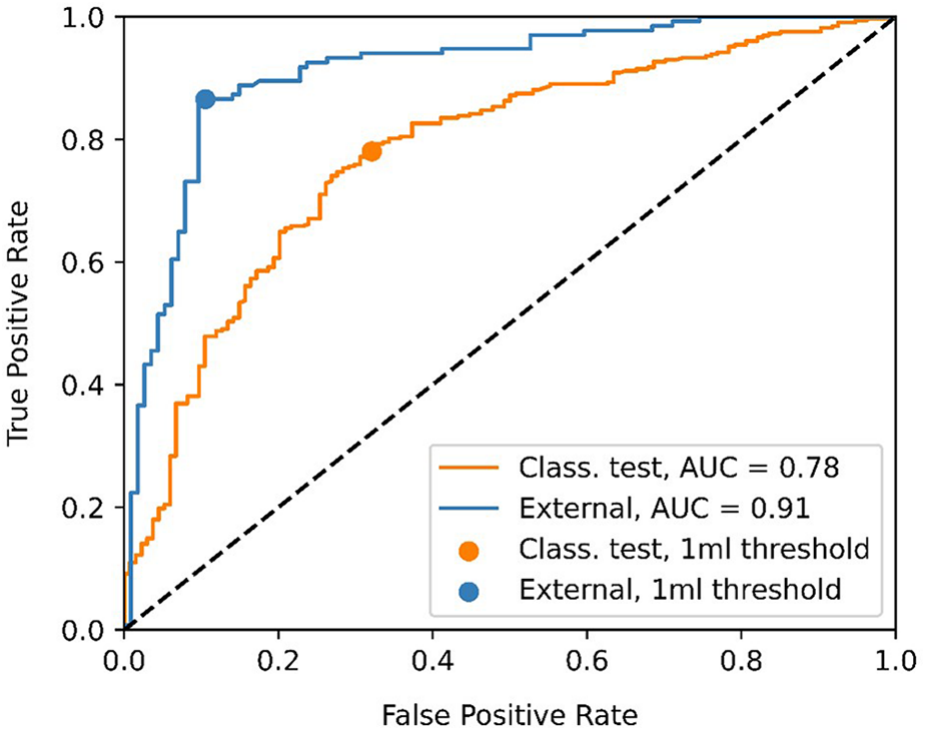
Receiver operating characteristic (ROC) curve showing the classification performance of the model at different enhancing tumor volume thresholds. The point closest to the 1 ml threshold is indicated as well as the area under the curve (AUC) for the classification test subset and the external dataset.

**Figure 5:**
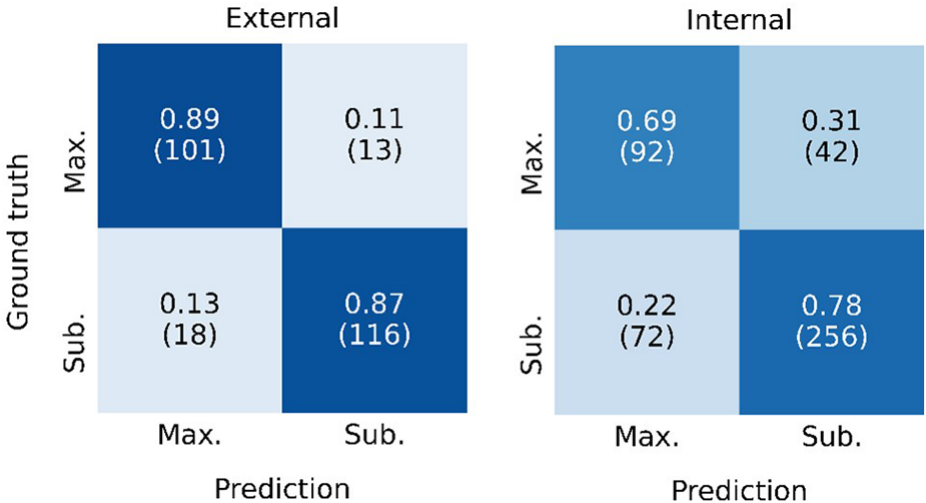
Confusion matrix showing the classification performance of the model at the 1 ml threshold for the external dataset (left) and the internal classification test subset (left). Maximal CET resection corresponds to a predicted volume <1 ml, larger predicted volumes are classified as submaximal CET resection. The rates are normalized over the ground truth classifications (rows). The number of patients in each category is given in parentheses.

Figure 4 shows the ROC curve for the EOR classification, corresponding to the internal classification subset and the external dataset. A positive classification denotes a submaximal CET resection. Our model achieved an AUC of 0.91 and 0.78 for the external dataset and the internal classification subset, respectively. The true vs. false positive rates at the 1 ml threshold are marked on the plot. The confusion matrices for the 1 ml threshold calculated for the classification test subset and the external dataset are given in Figure 5. On the external dataset, the model achieved a classification precision of 0.90 and a recall of 0.87 (*F*_1 _= 0.88), while on the internal classification subset we saw a precision of 0.86 and a recall of 0.78 (*F*_1 _= 0.82).

**Figure 6:**
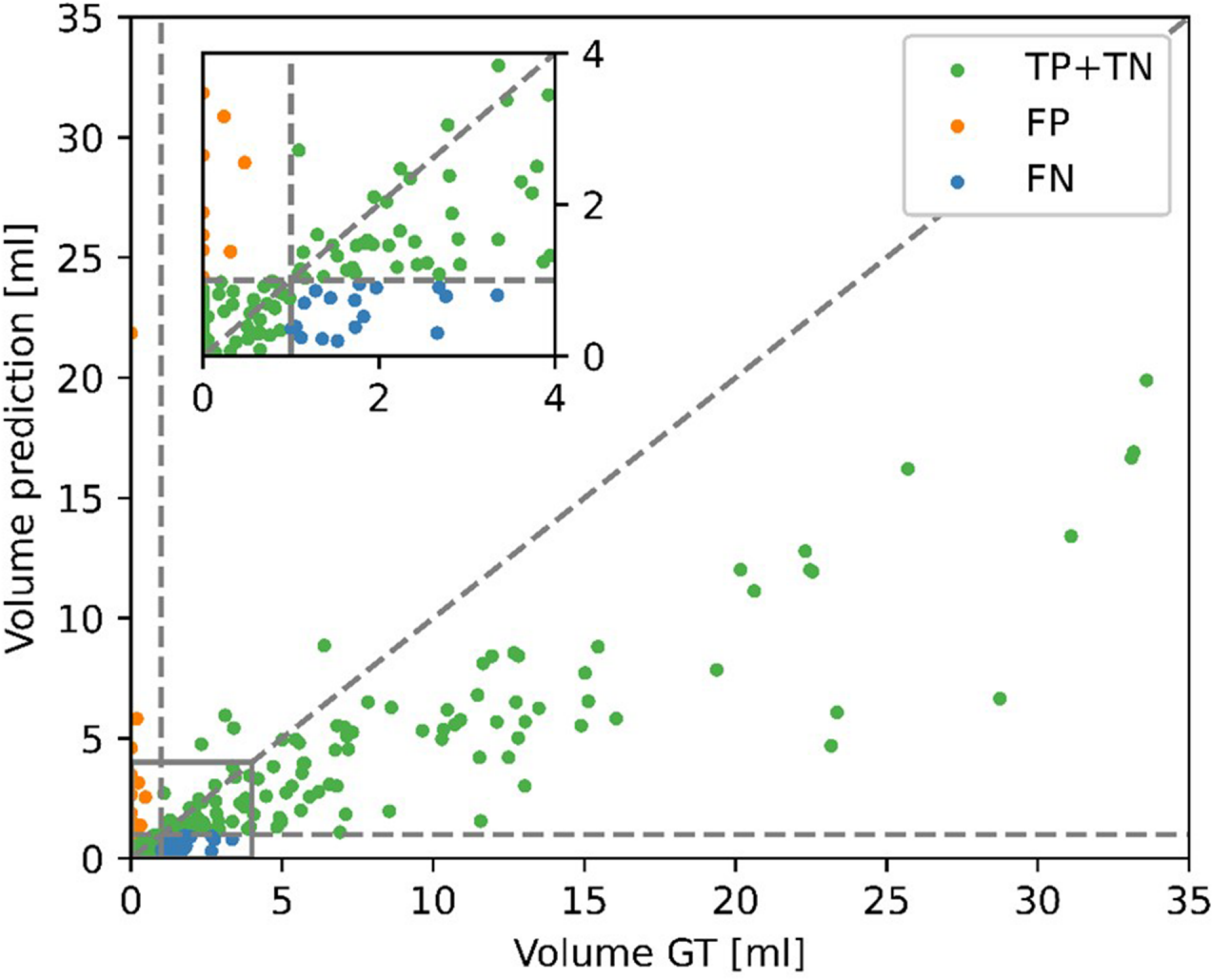
Contrast-enhancing tumor (CET) volumes in the predicted segmentations as a function of the CET volumes in the ground truth (GT) segmentations for the external dataset. Correct classifications (TP, true positive and TN, true negative), false positive (FP) and false negative (FN) are color-coded. A positive classification refers to submaximal CET resection, negative is maximal CET resection.

Given the disagreement between the mean predicted and GT volume, as seen in Table 2, the high classification performance of the model on the external dataset warranted further exploration. Figure 6 shows the predicted volumes as a function of the GT volumes. Most datapoints are below the diagonal line, meaning the model underestimates the volume, which is consistent with the lower average predicted volume shown in Table 2. However, all misclassified exams have GT volumes of <4 ml, as the underestimations for larger volumes are not of sufficient size to misclassify the exams. The model is more likely to misclassify exams with GT volumes close to 0 as opposed to GT volumes close to the 1 ml threshold. All false positive scans had a predicted volume of <0.5 ml.

### 3.5 Survival prediction

**Figure 7:**
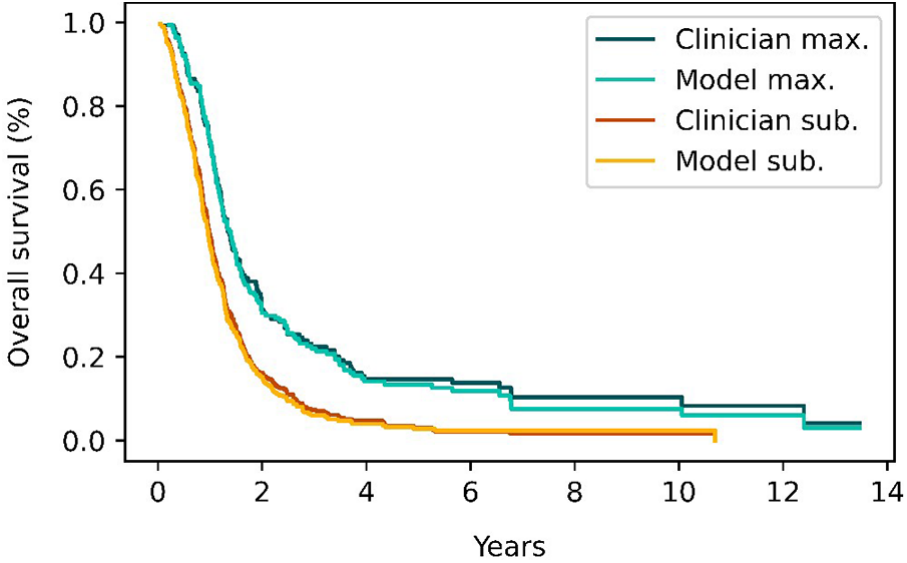
Survival curves for patients classified as having either maximal (red and orange curves) or submaximal (blue and cyan) enhancing tumor resection by either clinical evaluation or the model's classification. The survival curves from the model closely match the survival curves from clinician's classifications.

Figure 7 shows the Kaplan–Meier survival curves for patients in the classification test subset and stratified by the EOR determined by clinicians and the model. Median overall survival was 14.8 months (IQR: 10.3–29.2) vs. 16.6 months (IQR: 10.9–29.8) survival for the maximal CET resection groups as classified by clinicians and the model respectively. The corresponding values for the submaximal CET resection groups were 11.2 months (IQR: 6.7–17.5) vs. 10.3 months (IQR: 6.2–15.6). Although the differences were not significant (*p* = 0.93 and *p* = 0.41 for maximal and submaximal CET resection), the difference in median overall survival between the maximal and submaximal groups was larger when the classification was done by the model (6.3 months vs. 3.6 months).

### 3.6 Example cases

**Figure 8:**
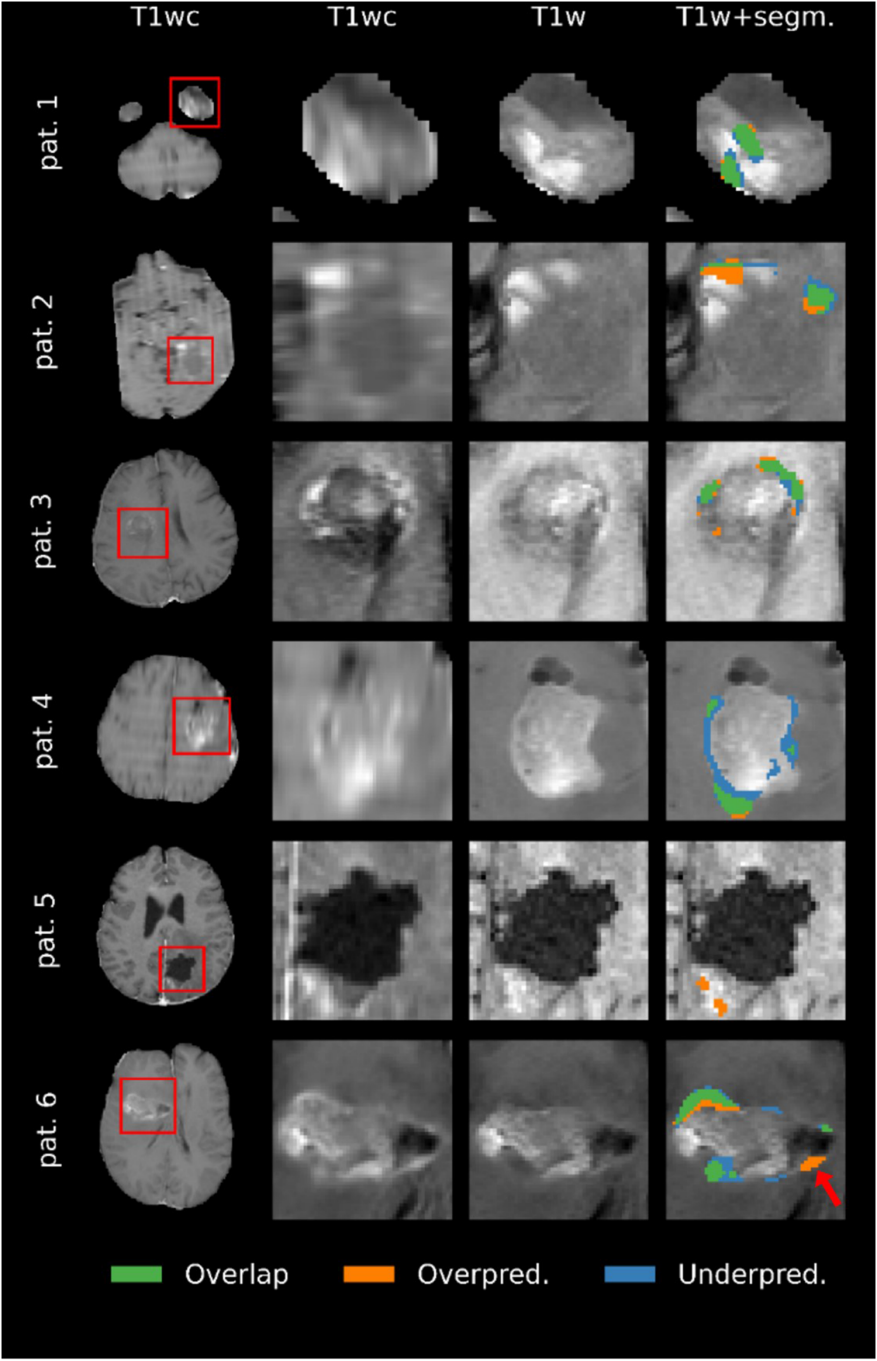
Comparison of the ground truth (GT) segmentations to the predicted segmentations for six patients. Overlap means that the voxel was segmented as enhancing tumor in both the prediction and the GT, overprediction indicates that a voxel is segmented in the prediction but not in the GT, and underpredicted voxels are segmented in the GT but not the prediction. Images are zoomed in to show all segmented voxels in the image. Note that the quality of the images reflects the resolution of the original MRI scans.

Representative examples of the predicted segmentations on the segmentation test subset are shown in Figure 8. The main failure mode of the model, as determined by the evaluating clinicians, was the misallocation of blood voxels being segmented as tumor. The model's overestimation in example patients 2 and 5 are examples of this, where it is likely that low-resolution data resulting in poor registration caused the model to mislabel blood as tumor tissue. The prediction in patient 1 was deemed likely be an example of the model mistaking blood for tumor. However, it is worth noting that the annotator had labeled the same area as tumor. Example patient 4 was particularly challenging due to the poor quality of the image. As a result, the evaluating clinicians were uncertain as to whether the area segmented as tumor in the GT but not in the model's prediction (blue in Figure 8) actually constitutes tumor tissue. Patients 3 and 6 were accurately predicted by the model, with some areas showing predictions that were deemed by the three evaluating clinicians to be better than the GT segmentation. Notably, the model's overestimation of patient 6′s scan (see arrow in figure) was deemed as tumor on closer inspection, despite the GT segmentation not including that region.

## 4 Discussion

The current study investigated the use of a U-Net-based deep-learning model to segment residual tumor on early post-operative MRI exams of patients with glioblastoma. This approach could help clinicians adhere to the latest guidelines by facilitating post-surgical tumor segmentations. We found that the model segments early post-surgical exams on par with expert annotators and is also capable of classifying the EOR of the CET.

Early post-operative glioblastoma is challenging to segment, as evidenced by Visser et al. in a study that shows a Jaccard score between expert annotators of only 0.33, corresponding to a Dice of 0.48 (9). With a mean Dice of 0.52 in the external dataset and 0.51 in the train/validation subset for the cases with a non-empty GT, the results show that the agreement between the predicted and the GT segmentations is comparable to the agreement between expert annotators as reported in literature. Note that the goal is not to achieve Dice scores higher than those between expert annotators, as this would imply overfitting to the annotator's preferences. Clinicians not involved in the annotation process scored the model's and the GT segmentations similarly, further indicating that these are comparable.

Although the model performs on par with expert annotators, the predicted segmentations are imperfect. Visualization of the predicted segmentations showed that most failures could be attributed to the model confusing blood products and tumor tissue, especially on low-resolution data with poor inter-scan registration between T1w and T1wc scans. These were also the cases where domain expert annotators often struggled. It is worth noting, however, that some of the errors in the GT segmentations may have been caused by a failure to correct errors in the preliminary segmentation provided to the annotator.

Following the newest guidelines, we used a 1 ml threshold to classify the predicted segmentations into either maximal or submaximal CET resection. Not surprisingly, we find that the model performs better in classifying the exams in the external dataset, where the GT classification was extracted using the same threshold, compared to the classification test subset where the GT classifications were set by clinicians following current clinical practice (CET present/not present). Using the 1 ml threshold, our model obtains a reasonable 0.90 precision and 0.87 recall. This is the case even though there is a substantial domain shift between the data the model is trained on in the external dataset, with an average GT volume of 1.77 ml vs. 4.40 ml respectively. A study by Helland et al. on early post-operative glioblastoma segmentation finds similar results, with their best-performing model trained on over 800 annotated exams obtaining 0.90 precision and 0.86 recall (23). While the results are not directly comparable, due to the use of different datasets and volume thresholds between the studies, they do caution that larger training datasets do not necessarily lead to better predictions. In an era when lack of reproducibility of AI models plagues the medical field, we believe that showing that two independently developed models trained on different datasets give similar results helps strengthen confidence in the use of AI in this clinical context.

When used to stratify patients according to their CET EOR, the model performs at least as well as the clinical classification in predicting overall survival. In fact, using the model leads to better stratification, although this result is not statistically significant: The difference in median overall survival between patients with maximal and submaximal EOR is 6.3 months, compared to 3.6 months when using the clinical classification. Using overall survival as a benchmark provides an objective method to assess the model's performance. This is particularly useful when working on segmentation of glioblastoma in MR images taken shortly after surgery, when the inter-rater disagreement is considerable. Moreover, this finding emphasizes the clinical relevance of the model's classifications, since stratifying patients according to their overall survival is one of the main applications of EOR classifications. We hope that our results concerning the difference in survival between EOR groups can serve as a benchmark for future studies.

### 4.1 Limitations and future work

While the predicted segmentations are, overall, on par with expert annotators, the limited size of the training dataset increases the risk of the model failing to segment tumors in the presence of pathologies or features (for example, artifacts) it has not encountered during training. As with any deep-learning model, it is imperative that clinicians review the model's predictions when used in clinical practice. Of the errors we encountered, most appear to be caused by a failure to distinguish between tumor tissue and blood products under certain conditions, like subpar registration. Higher resolution data, or in lieu of this, data augmentation simulating poor registration by introducing offsets between the volumes could improve segmentations. There is also a tendency for the model to underestimate tumor volumes in the external dataset, particularly in the case of large tumors, suggesting that the model may be suboptimal for reliable volume analysis. The cause of this bias is likely the considerably lower mean GT volume in the training dataset compared to the external dataset, which might be due to differing annotation practices. When using the 1 ml threshold as proposed in the latest guidelines, the volume bias does not substantially affect the resulting EOR classification. However, using lower thresholds will lead to higher false positive rates, and this model should not be used to perform EOR classification using other thresholds without prior validation.

Another limitation is that the GTs are annotated by a single radiologist. Using majority voting among multiple annotators could improve the reliability of the model, but not without significantly increasing the resources needed. Alternatively, capturing the annotator's confidence in each segmentation and using it to train a confidence-aware network has been shown to improve predictions in post-operative scans taken at later dates (16), and could also be helpful for early post-operative segmentation given the uncertainty faced by the annotators. An additional limitation is that our model only segments residual ET, and not the non-enhancing tumor-infiltrated tissue. The latest RANO guidelines conclude that additional resection of the non-enhancing tumor provides some benefits over maximal CET resection alone (7). Hence, further work is needed to train segmentation models capable of classifying the EOR of the non-enhancing tumor.

## 5 Conclusion

Our trained deep learning model was capable of segmenting residual CET on post-operative MRI exams with a performance comparable to the interrater agreement between expert annotators as measured by the Dice score. On the internal test dataset, the clinical value of the segmentations was corroborated by the subjective evaluations of three clinicians who rated the predicted segmentations at a similar level as the GT segmentations. However, the model substantially underestimated the tumor volumes in the external dataset, suggesting it may be suboptimal for reliable volume analysis. Despite this volume bias, when used to classify the CET EOR the model achieved precision/recall scores of 0.90/0.87 on the external dataset and 0.86/0.78 on the internal dataset. Moreover, we show that stratification of patients based on the model's predictions has at least the same prognostic value as when done by clinicians. This work may help streamline the introduction of the newest RANO guidelines into clinical practice by providing accurate and replicable CET EOR classifications without adding to radiologists’ workload.

## Data Availability

The datasets presented in this article are not readily available because of patient privacy protection concerns. However, the data presented in this study are available upon reasonable request from the corresponding author. Requests to access the datasets should be directed to lidialuquef@gmail.com.

## Footnotes

^1^ https://github.com/lidialuq/resect-glio

^2^ https://github.com/ANTsX/ANTs

^3^ http://www.itksnap.org

^4^ https://monai.io/

^5^We found that increasing the weighting of the unsupervised component of the loss improved the validation score. To that end, the weight parameter φ was changed to 2.
